# Nectar feeding beyond the tongue: hummingbirds drink using phase-shifted bill opening, flexible tongue flaps and wringing at the tips

**DOI:** 10.1242/jeb.245074

**Published:** 2023-04-03

**Authors:** Alejandro Rico-Guevara, Kristiina J. Hurme, Margaret A. Rubega, David Cuban

**Affiliations:** ^1^ Department of Biology, University of Washington, Seattle, WA 98195, USA; ^2^ Burke Museum of Natural History and Culture, University of Washington, Seattle, WA 98105, USA; ^3^ Department of Ecology and Evolutionary Biology, University of Connecticut, Storrs, CT 06269, USA

**Keywords:** Biomechanics, Drinking, Feeding mechanisms, Flow visualization, Fluid transport, Nectarivory

## Abstract

Hummingbirds are the most speciose group of vertebrate nectarivores and exhibit striking bill variation in association with their floral food sources. To explicitly link comparative feeding biomechanics to hummingbird ecology, deciphering how they move nectar from the tongue to the throat is as important as understanding how this liquid is collected. We employed synced, orthogonally positioned, high-speed cameras to describe the bill movements, and backlight filming to track tongue and nectar displacements intraorally. We reveal that the tongue base plays a central role in fluid handling, and that the bill is neither just a passive vehicle taking the tongue inside the flower nor a static tube for the nectar to flow into the throat. Instead, we show that the bill is actually a dynamic device with an unexpected pattern of opening and closing of its tip and base. We describe three complementary mechanisms: (1) distal wringing: the tongue is wrung out as soon as it is retracted and upon protrusion, near the bill tip where the intraoral capacity is decreased when the bill tips are closed; (2) tongue raking: the nectar filling the intraoral cavity is moved mouthwards by the tongue base, leveraging flexible flaps, upon retraction; (3) basal expansion: as more nectar is released into the oral cavity, the bill base is open (phase-shifted from the tip opening), increasing the intraoral capacity to facilitate nectar flow towards the throat.

## Teaser

**Summary:** Live visualization of hummingbird bill movements and intraoral space reveals how they transfer nectar from the tongue to the throat – the eco-evolutionary implications of these findings are discussed.

## INTRODUCTION

Hummingbirds evolved to drink from flowers well enough to live off of small volumes of nectar scattered over the landscape, and the resulting adaptations – hovering flight, high metabolism, body size reduction – shaped and were shaped by their novel lifestyle and biology. Co-evolution between floral shape and bill morphology has produced a family of birds, Trochilidae, with elongate bills that range from slightly recurved to strongly decurved (up to over 100 deg; Boehm et al., 2022). As the consensus is that the evolutionary trajectory and remarkable biology of hummingbirds arises from feeding on flowers (Rico-Guevara et al., 2021; Leimberger et al., 2022), research on how they drink the floral nectar is pivotal to understanding their evolution. Most of the research on this front has focused on how their bills fit in the flowers (the ‘bill–corolla matching hypothesis’, reviewed in Rico-Guevara et al., 2021), and on how their specialized tongues collect the reward (reviewed in Rico-Guevara et al., 2019a), with connections to evolutionary implications such as nectar concentrations in flowers (Heyneman, 1983; Rico-Guevara and Rubega, 2012). The hummingbird tongue bifurcates distally (i.e. bifid; Darwin, 1841), ending in two parallel semi-cylindrical tubes or grooves, formed by rolling of the thin tongue margin (Lucas, 1891; Scharnke, 1931). Their tubular tongues (Martin, 1833; Sundevall, 1872; Gadow, 1883) are the main structures involved in nectar collection, but their functioning has been revealed to be quite different from what was historically thought (summarized by Nicolson and Fleming, 2014; Rico-Guevara et al., 2019a); moreover, liquid pickup by the tongue is only the first step in a series of intricate events during nectar drinking. As the limiting step of this feeding process is unknown, understanding nectar drinking mechanics beyond its collection would take the field closer to solving long-standing questions on the animal (e.g. why is it that not all hummingbirds have long bills?) and plant side (e.g. are there concentration/volume optima for particular pollinators?).

Once the tongue is loaded, and retracted into the hummingbird's mouth, different mechanisms are involved in the middle stage – offloading – and the final stage – intraoral transport of nectar up the elongate bill, and swallowing of nectar (stages *sensu* Cuban et al., 2022). These stages are poorly known and the surrounding hypotheses come mostly from morphological studies. Lucas (1891) pointed out that no vacuum can be formed at the base of the tongue, and Scharnke (1931) and Weymouth et al. (1964) emphasized that there is no connection between the distal semi-cylindrical grooves and the tongue base (Gould, 1861); therefore, the tongue cannot function as a ‘soda straw’. Drinking liquid through a closed tube, or straw, works by generating a region of lower pressure inside the mouth relative to the ambient pressure (which the liquid of the drink is subjected to); thus, the pressure differential results in a force that pushes (from the outside) the fluid through the straw (Cuban et al., 2022). The hummingbird tongue grooves have dorsal slits and flexible walls that prevent the formation of a vacuum and yield to collapsing of the structures, respectively (Kingsolver and Daniel, 1983; Rico-Guevara, 2017), yet this does not preclude potential flow of a liquid column through them if the fluid is exposed to other forces such as basal pumping (e.g. as in the lepidopteran proboscis; Monaenkova et al., 2012, Kornev et al., 2017). Hainsworth (1973) suggested that the grooves transport a relatively small amount of liquid from the nectar reservoir to the bill, and hypothesized that a substantial volume of nectar was channelled into the beak along the sides and on top of the tongue, via adhesion (as in dog lapping; Crompton and Musinsky, 2011), but his inferences could have been misled by low filming speeds (Ewald and Williams, 1982). High-speed videography has shown fluid capture at the tongue tips (Rico-Guevara and Rubega, 2011) and elastic filling of the rest of the tongue grooves (Rico-Guevara et al., 2015). Hence, the nectar needs to be wrung out of the tongue grooves (Rico-Guevara and Rubega, 2017) to continue its journey to the throat.

The actions of the bill and tongue during nectar consumption are cloaked by the floral corolla, but they can be exposed using faux transparent flowers (reviewed in Rico-Guevara et al., 2019a). Nonetheless, the transport of the nectar from the tongue to the pharynx is still concealed inside the keratinized and often melanized beak, and hence thus far it has been a ‘black box’. As nectar extraction must often work against gravity (or without its aid), multiple authors have proposed intraoral transport hypotheses (reviewed by Rico-Guevara, 2014; Cuban et al., 2022); briefly summarized as follows. (1) Cohesive pulling: when a structure moves proximally, it uses forces between water molecules to pull fluid attached to its surface (e.g. the tongue retracts, pulling adhered nectar), analogous to dragging a drop over a leaf with your finger. (2) Hermetic suction: a negative pressure differential is generated through the expansion of an air-tight cavity (e.g. tongue lowering while in contact with the roof of the mouth or separation of the jaws), like when sucking through a straw. (3) Cohesive suction: similar to hermetic suction except there is no need for an air-tight seal (e.g. bill base expanding with a gap on the sides); as in hermetic suction, cohesive forces linking water molecules draw liquid into a cavity, already filled with fluid, as it expands (you can also do this with a straw). (4) Hydraulic displacement: proximal flow driven by the introduction of additional liquid at the tip (e.g. an aliquot of nectar offloaded at the bill tip will be pushed backwards by an incoming aliquot taking its place), analogous to bowling balls pushing each other on a return system. (5) Capillarity: in the same way as water fills small glass tubes when in contact with the surface, if the feeding apparatus forms a bridge between the tongue and the inside of the bill, capillarity would generate a meniscus of nectar pulling the nectar proximally.

Beyond the specific nectar-feeding hypotheses above, there are more general avian feeding mechanisms to consider. Generally, food transport is aided by gravity and/or the tongue base, which in most birds exhibits a structure called the ‘papillary crest’ (reviewed in Parchami et al., 2010; Erdoğan and Iwasaki, 2014) and is triangular, spiny and directed proximally, helping to move food items towards the throat (Rico-Guevara et al., 2019b). In hummingbirds, the papillary crest is reduced to two flexible flaps on either side of the base of the tongue, arranged in a ‘V’ configuration and directed backwards and upwards, which are also called ‘tongue wings’ (i.e. alae linguae; Weymouth et al., 1964; see fig. 7C in Rico-Guevara, 2017). Transporting liquid foods, or drinking, in birds is distinct from that in many other vertebrates in that, with a few notable exceptions, birds have short, simple tongues invested with little flesh or musculature (Homberger, 1999; Erdoğan and Iwasaki, 2014). Thus, neither hydrostatic pumping, achieved by muscular compression and elongation, nor lapping, made possible by tongue extension (e.g. Crompton and Musinsky, 2011), is possible for most birds. Only a few avian taxa have been reported to drink while keeping their heads down, including pigeons and mousebirds (reviewed in Homberger, 2017; Rico-Guevara et al., 2019b). Instead, scooping water into the lower jaw cavity then transporting it to the pharynx by tipping the head up is widespread in birds. This is true even in shorebirds, which also have narrow, elongate beaks (M.A.R., personal observation); although a long list of shorebirds have been shown to use surface tension to transport food up the beak using water droplets (Rubega, 1997; Estrella et al., 2007), when they drink, they scoop and head tip as most other birds do.

Surface tension transport in shorebirds is unusual in the sense that the oral transport mechanism is visible externally. This mechanism takes advantage of differential capillary forces acting on the front and rear menisci of a liquid droplet/column, created by a larger distal opening (and a corresponding smaller proximal opening) of the bill, to move the liquid towards the bill base (Rubega, 1997; Prakash et al., 2008). For this mechanism to work, the angle of bill opening needs to be large enough to move the droplet (stuck to both the upper and lower bill) backwards, and in long-billed birds, such as shorebirds, this translates into bills with visible separation of the jaws all the way to the bill base, in which the motion of the fluid can be easily studied. We do not observe this kind of extensive jaw separation in hummingbirds during nectar drinking (as seen during insect capture; Yanega and Rubega, 2004), so the same surface tension mechanism of shorebirds is discarded, which also means that *in vivo* intraoral visualization of the motion of the tongue and fluid through the bill keratin is required. This has been accomplished in the past through fluoroscopy in conjunction with lead markers, which for a long time was only available at low recording speeds (48 frames s^−1^; e.g. Zweers et al., 1977, Zweers, 1982, Kooloos and Zweers, 1989). Modern high-speed fluoroscopy has been used on intraoral fluid transport of larger animals (dogs, 500 frames s^−1^; Crompton and Musinsky, 2011), and it can achieve very high spatiotemporal resolution (3000 frames s^−1^; Lucas et al., 2018). X-ray reconstruction of moving morphology (XROMM; e.g. Brainerd et al., 2010) has enhanced the study of feeding, offering high-speed capability (250 frames s^−1^; Dawson et al., 2011) and 3D reconstruction through orthogonal views. However, current XROMM systems do not provide the millimetric scale resolution required to study nectar intraoral transport in hummingbirds. Synchrotron imaging has also been used to study drinking in insects, but its micrometric resolution is often associated with small fields of view and low frame rates (e.g. 30 frames s^−1^; Kim et al., 2011; Lee et al., 2014; Kikuchi et al., 2018), and lateral views only. Additionally, these X-ray setups are not portable, impart high radiation exposures, and require the addition of electron-dense substances, which further remove the results from the inferences about natural function. To surmount these challenges and unveil the nectar transport mechanics, we combined external high-speed videography and field-friendly backlit filming, achieving intraoral visualization, thus illuminating the understanding of drinking in hummingbirds.

## MATERIALS AND METHODS

### External measurements

**Fig. 1.**
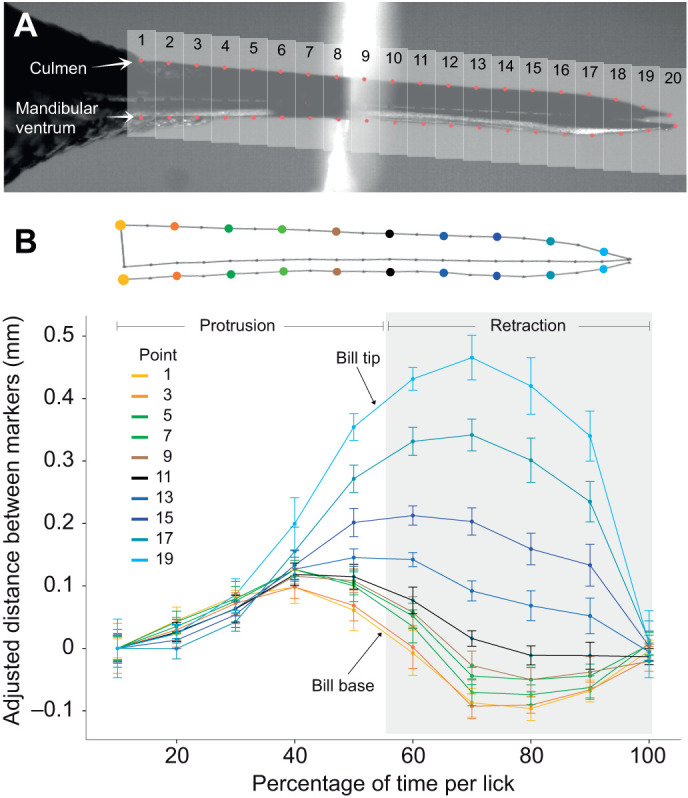
**Bill kinematics through the lick cycle in hummingbirds.** (A) Semi-landmark placement scheme for dorso-ventral motion analysis. Still picture taken from a high-speed video of an Anna's hummingbird (*Calypte anna*) drinking nectar. Bill regions with corresponding semi-landmarks are numbered. (B) Distance between culmen and ventrum at each point along the bill, as a function of time. In order to control for the change in thickness along the bill, we adjusted the initial separation between each pair of markers to zero at the first time step. Given that the duration of each lick was different, we standardized every time step to a percentage of time throughout the lick cycle. Note that the opening at the distal regions is larger than the decoupling at the basal region. Means of 10 licks ±1 s.d. are shown. The grey shading indicates the portion of the lick cycle where tongue retraction occurred.

We performed a general characterization of the hummingbird feeding apparatus kinematics while nectar drinking as this was unknown. External videos of the bill allowed us to assess potential lateral bill base expansion (ventral and/or dorsal) and bill-base dorsoventral separation (upper and lower bill separating at the base) in order to test the hypotheses involving bill base expansion (Cuban et al., 2022). We filmed free-living hummingbirds trained to drink nectar from artificial feeders with clear sides (e.g. Rico-Guevara and Rubega, 2011; Rico-Guevara et al., 2015) to study bill and tongue kinematics. We worked in Colombia, Ecuador and the USA (all private locations with the permission of their owners). We filmed adult birds (judging by the absence of visible corrugations at the bill base) drinking while hovering, with high-speed cameras (TroubleShooter HR, Fastec Imaging, 1000 frames s^−1^, 1280×512 pixels) coupled to macro lenses (Nikon 105 mm f/2.8 VR). We recorded 24 individuals belonging to seven genera and species, and to three out of the nine currently recognized clades in the family, collecting data on bill movements along tongue reciprocation cycles (see Supplementary Materials and Methods, Tables S1–S3). We present detailed data on bill kinematics as well as the timing of tongue reciprocation, for one individual per species. This sampling scheme prevented us from studying intraspecific variability; therefore, we focus only on interspecific differences, with the caveat that the results could be biased as a result of idiosyncratic variation at the individual and sex levels (which warrant their own studies). When possible, tongue length at each frame was calculated as the sum of the distances between each point along the tongue contour using the distance measurement analysis function in tpsDIG2 (Rohlf, 2010). When tongue length measurements were not possible, we noted its kinematic state as protruding, retracting or switching directions/not moving, by comparing a series of consecutive frames until the kinematic state was determined. For every bird, we randomly selected 10 licks from different foraging bouts; videos (in lateral view) were converted into a series of image files (e.g. Fig. 1A). All images in each sequence were digitally enhanced equally by consistently adjusting the contrast and brightness using ImageJ 1.45p (Schneider et al., 2012), in order to maximize the visibility of the bill contours. We assessed the separation between the maxilla and mandible, along the different bill regions throughout the lick cycle, by obtaining the coordinates for bill profile semi-landmarks (details in Supplementary Materials and Methods) and calculating the distance between each corresponding pair of points in the culmen and ventrum (Fig. 1A). By taking the maximum and minimum separation distance at each point-pair per lick, and averaging across licks, we obtained the range per culmen–mandibular ventrum point-pair along the bill.

In order to quantitatively assess differences in the ranges of upper to lower bill separation, we followed two approaches. First, we modelled ranges of separation as a function of species, with a random intercept of culmen–ventrum point-pair along the bill length. As bill semi-landmarks (Fig. 1A) are spatially autocorrelated in one dimension, we included a first-order autoregressive covariance structure on point-pairs along the bill grouped by species. We ran generalized linear mixed models (GLMMs) for both the ranges of separation in millimetres and normalized (proportional to maximum point-pair range per species) using the package ‘glmmTMB’, visualizing the interspecific differences through forest plots, in R version 4.1.3 (http://www.R-project.org/). Second, we constructed a generalized additive model (GAM) with ranges of separation as a response variable, species as a fixed factor and separate smoothers for each species with *k*=20 (20 nodes) using the package ‘mgcv’ in R version 4.1.3 (https://CRAN.R-project.org/package=mgcv). We then calculated the differences between each pairwise set of species smoothers, along with the confidence interval of the differences (after Rose et al., 2012), in order to assess not only whether species are different but also where along the bill the 95% confidence intervals do not overlap.

### Intraoral measurements

Visualization/tracking of the nectar and the tongue position inside the bill is crucial to test hypotheses involving different fluid dynamics forces (Cuban et al., 2022) and their potential actuators. Intraoral fluid tracking was possible for the rufous-tailed hummingbird (*Amazilia tzacatl*) because of its red bill (lacking heavy melanin deposition along most of its length), which makes it translucent when illuminated from behind. We positioned a flashlight (Energizer^®^ Aluminium Alloy Waterproof Lithium LED Flashlight) below the feeder, and prevented the hummingbird from noticing the light by using an opaque cardboard barrier with a hole as a corolla entrance. The inclination of the flashlight was intended to maximize light transmission through the keratin, in order to visualize their bills as translucent in the dorsal plane. When properly lit, both the tongue base and the nectar front (fluid meniscus) appeared as shadows inside the backlit bill. We collected macro high-speed videos (as described above, but at 500 frames s^−1^ to increase recording time) of orthogonal views (dorsal and lateral) and calculated the position of the tongue base as the distance from the bill tip to the midpoint where the tongue base was located between the bill edges. We also measured the distance from the bill tip to the tongue tip outside the bill in both the lateral and dorsal views, for all licks. We chose four lick sequences in which we were able to accurately visualize the tongue base with high confidence through the entire lick cycle, and measured the position of the tongue base as the Euclidean distance from the bill tip. We compared the tongue base tracking to the tongue tip tracking by correlating the distance measurements to fully reconstruct the tongue movements in six additional licks (for a total of 10 lick cycles). Assuming that the tongue does not change in length (i.e. it is not longitudinally stretchable; Weymouth et al., 1964; Hainsworth, 1973) and calculating the total tongue length in the sequences in which it was visualized with high confidence, we calculated the approximate position of the tongue base for the sequences in which it was difficult to visualize. We did this using the tongue protrusion distances measured in lateral and dorsal views to subtract them from the tongue total length; we used the remainder as the distance from the bill tip to the tongue base inside the bill. The complete retraction of the tongue into the bill was used as the minimum base distance and was the last frame of each sequence; we started the next sequence when the tongue began protruding again.

In the dorsal-view backlit videos of *A. tzacatl*, we tracked the progression of nectar intake through the intraoral cavity. Nectar was visible in the lit bill as a shadowy mass (liquid column) with a distinct edge, the nectar front/meniscus. Intraoral flow was measured as meniscal position within the bill at a 500 Hz sampling rate from the time at which a nectar meniscus was initially visible in the most distal point within the bill, and continuing through its proximal progression to the bill base. In each image, the meniscus midpoint between the bill edges was sampled. To do this, two reference points were placed where the fluid came into contact with the inner bill walls; we traced a line between the reference points and resampled it to find a midpoint equidistant to each reference point. The midpoint of the meniscus was used to track the nectar flow using the maxillary bill tip as the reference point.

All filming activities were reviewed and authorized by the Institutional Animal Care and Use Committee at the University of Washington (UW) and at the University of Connecticut (UConn); UW IACUC 4498-01, 4498-03, and UConn Exemption Number E09-010.

## RESULTS

### External measurements

**Fig. 2.**
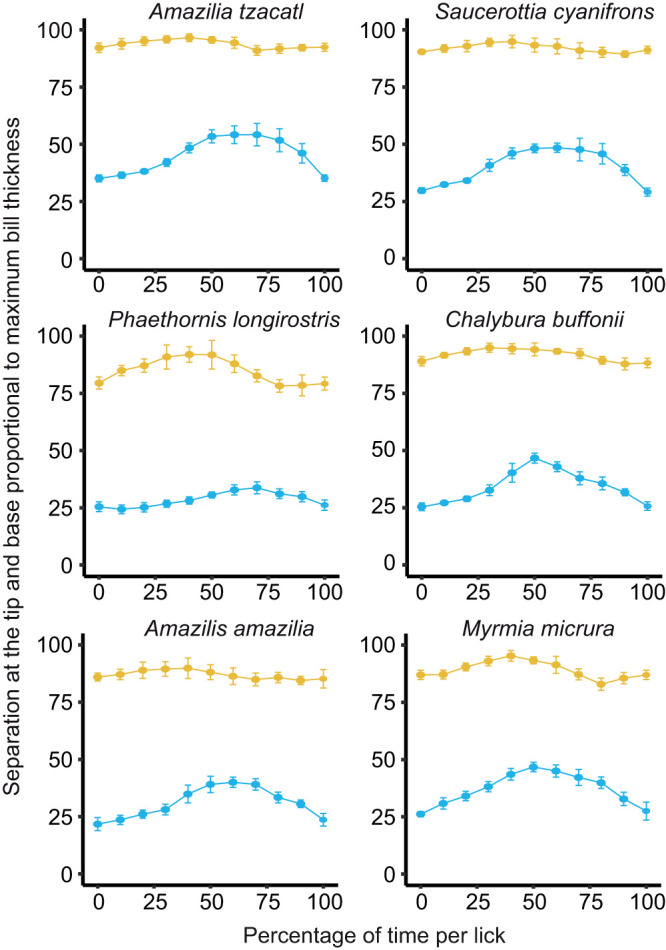
**Separation distance between culmen and mandibular ventrum point-pairs through the lick cycle for six species of hummingbirds.** Separation distance is given as a proportion of bill thickness against the percentage of time per lick. Only two selected point-pairs are shown: point-pair 2 (yellow lines at the top of each graph, corresponding to the bill base) and point-pair 19 (light blue lines below the yellow ones, corresponding to the bill tip). We do not present data on Anna's hummingbird because it follows the same trend (Fig. 1) and a more detailed comparative analysis is presented elsewhere (Fig. 3; Figs S1, S3, S4). Using 10 randomly selected licks from different foraging bouts, we obtained a mean wave pattern (±s.d.) for each point-pair per species.

We did not observe lateral expansion of the bill base (e.g. ventral, Movie 1; dorsal, Movie 2); however, we do not discard the possibility of flexible deformation of the intraramal skin and throat which we did not quantify. In lateral view, a general expectation for bill opening is set by their lever mechanisms of opening/closing of the entire upper and lower jaws hinging upon the naso-frontal and quadrato-mandibular articulations, respectively (e.g. Rico-Guevara et al., 2019b). Considering basal hinges in the bill, the general expectation is that opening/closing movements at the tip will be accompanied by in-phase synchronized (same period) opening/closing motions of the base, but of smaller amplitude (like opening/closing a drawing compass or kitchen tongs). In our lateral videos, we did not observe the expected pattern of opening and closing for avian bills noted above. Instead, we found synchronous phase-shifted (out of phase) opening and closing between the bill base and the bill tip, while the middle portion of the bill stayed relatively closed (Fig. 1B; Fig. S1). The separation of bill tips reached its maximum right before the separation of the upper and lower jaws at the bill base reached its minimum (phase lag ∼20–30%; Figs 1B and 2; Fig. S1). This phase offset, however, varied to a large degree among species and individuals (Tables S2, S3), but on no occasion did we find in-phase bill base-tip opening/closing; the phase-shifted pattern we report here was ubiquitous to all hummingbirds studied.

**Fig. 3.**
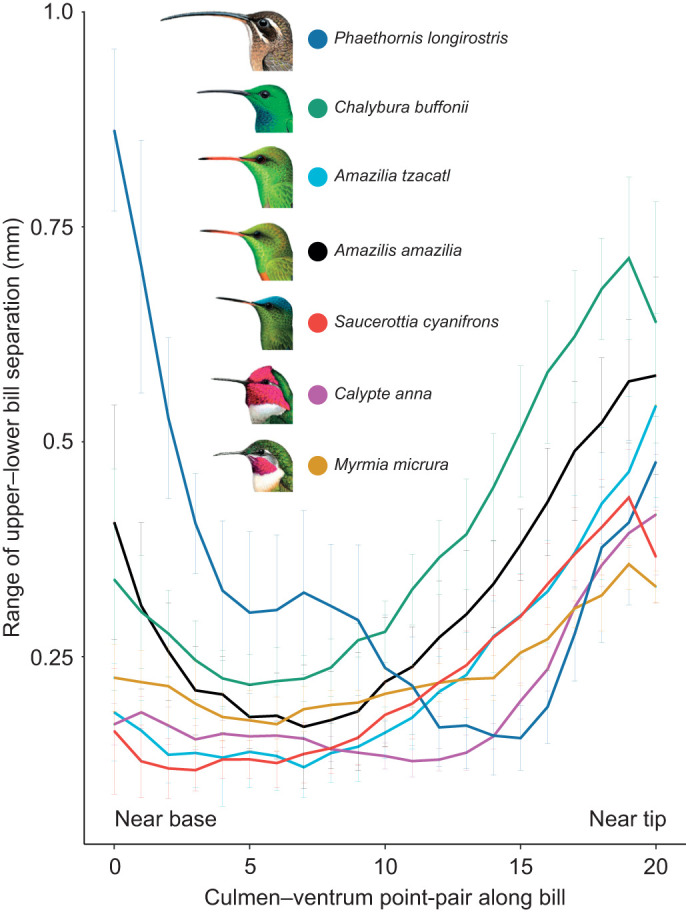
**Range of separation between dorsal upper bill profile and ventral lower bill profile for seven species of hummingbirds.** We obtained the range per culmen–mandibular ventrum point-pair by subtracting the maximum and minimum separation values along the lick cycle. We used 10 randomly selected licks from different foraging bouts and report mean patterns (±s.d.) per species. Species are organized in phylogenetic order by clade [from top to bottom: hermits (1), emeralds (2–5) and bees (6,7); Table S1], and within clade by bill length. Head profiles are from Birds of the World (Cornell Lab of Ornithology, https://birdsoftheworld.org/bow/home; used with permission). We provide statistical analyses testing the differences among these curves in Figs S3 and S4.

To facilitate comparisons across species, we analysed the range of separation (maximum minus minimum distance between markers, e.g. Fig. 1B) per point-pair along the lick cycle – collapsing the temporal component – presenting only one line per species, in absolute (Fig. 3) and proportional (Fig. S1) terms. We observed a similar pattern for all the species studied (Table S1); all exhibited a region in which the bill separates less than at the base or the tips. Also, there was generally more variability in the amount of separation at the extremes of the bill than in the middle of the bill, which could result from reduced error given that the amplitude/speed of movement was lower near the middle of the bill and/or because the middle region serves as a hinge where the separation is kept to the minimum and limited by the ‘occlusion’ of the upper and lower bill (internal abutting of the mandibular tomia against the roof of the mouth). It is worth noting that the species with the shortest bill (short-tailed woodstar, *Myrmia micrura*; Table S1) showed increased separation in this middle bill zone when compared with the other species in relative (Fig. S1) and, to a lesser degree, absolute terms (Fig. 3). Also, the species with the longest bill (long-billed hermit, *Phaethornis longirostris*) showed the largest separation of the bill base – but not of the bill tip – among the species studied, both relatively and absolutely (range of separation curves in Fig. 3; Fig. S1).

With the GLMMs with 1D spatial autocorrelation (see Materials and Methods), we found significant interspecific differences in the range of separation curves when using the raw data in millimetres (Fig. 3; Fig. S3A), but not with the normalized data (Figs S1, S3B). When comparing the range of separation along the bill in millimetres. Using the long-billed hermit (40 mm-long bill) as reference, the three species with the shortest bills (∼16 mm long; Table S1) were the ones with significant differences (Fig. S3A). This result highlights the influence of bill length in the separation kinematics, which results in higher absolute separation overall (potentially needed to move larger amounts of liquid through longer bills). When removing the effect of size through normalization, the lack of significant differences indicates that the general pattern – larger separation at the extremes of the bill than in the middle – applies to all species studied. From the GAM approach, we obtained all the pairwise comparisons, which can be used to statistically assess qualitative differences noted in the range of separation curves between species (Fig. S4). For example, when two separation curves are ‘right next to each other’ – less than ∼0.1 mm – or visibly overlapping (such as *Amazilia tzacatl*–*Saucerottia cyanifrons*; Fig. 2) the confidence intervals overlap (Fig. S4). The differences between ‘GAM smoothers’ are needed only when in doubt about large variation at a given point along the bill. Another noteworthy difference between the long-billed hermit and the rest is that at around three-quarters of the bill length (point-pair 15), there is a larger difference than at the tip (point-pair 20) with the other species (Figs S1, S4). The increase towards large separation at the tip (that in the hermit occurs between markers 15 and 16) starts more proximally in the rest of the species (ranging from marker 6 to 12), but taking into consideration that the other species have bills only about 40–60% of the Hermit's bill length (Table S1), such an increase actually occurs within the same range of absolute distances from the bill tip as in the rest of the species.

### Intraoral measurements

**Fig. 4.**
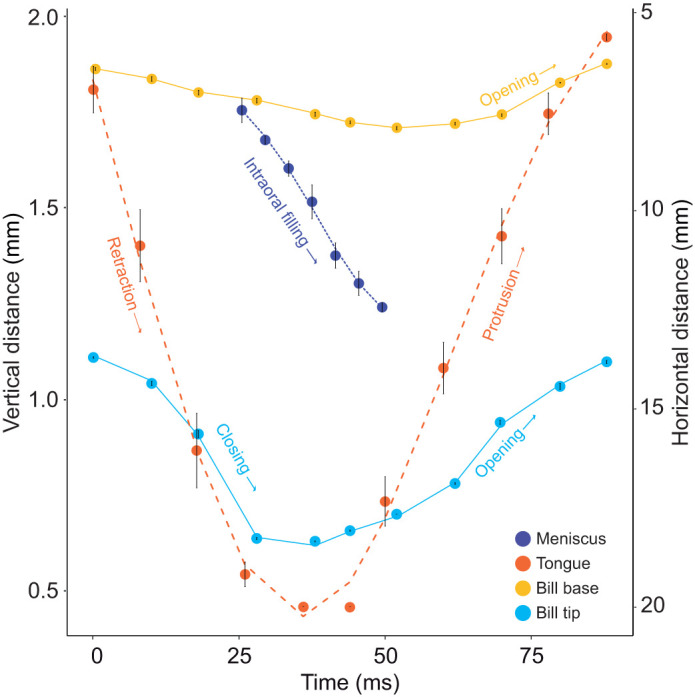
**Bill and tongue movements in relation to intraoral flow in a rufous-tailed hummingbird (*Amazilia tzacatl*)*.*** Left *y*-axis: dorso-ventral separation at the bill base (yellow) and at the bill tip (light blue). Right *y*-axis: horizontal distance from the bill tip to the bill base. Inferred motion of the tongue base (orange) varies from near 5 (maximum protrusion) to near 20 (tongue entirely inside the bill). Intraoral flow of nectar (in indigo), measured as the distance between the proximal nectar meniscus and the bill tip, was only trackable behind the black distal region (about 7 mm from the tip; Fig. 3). Mean (±s.d.) data from three consecutive licks (Fig. S2); interpolated lines were generated using quintic smoothing splines (package ‘npreg’ in R; https://CRAN.R-project.org/package=npreg).

**Fig. 5.**
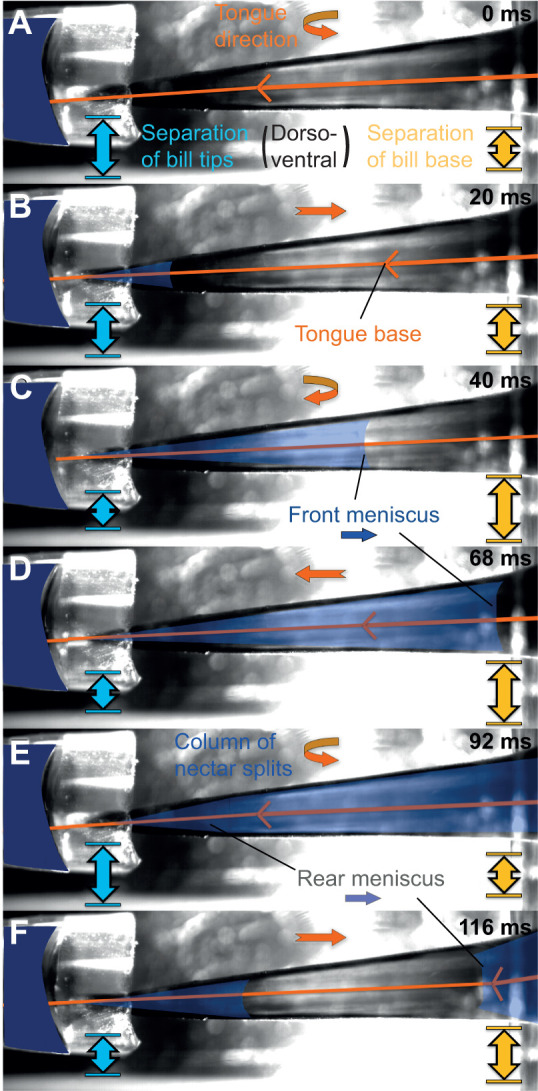
**Frames from a high-speed video of a rufous-tailed hummingbird (*A. tzacatl*) drinking nectar.** The dorsal-view sequence was captured through backlit filming yielding a ‘see-through’ bill and contains one and a half lick cycles starting with an empty oral cavity. Individual elements are indicated: tongue (orange) in the middle of the black outlined bill, tongue wings (‘V’ shape) and nectar (indigo shading). Straight orange arrows indicate the direction of the tongue movement (pointing to the left: protrusion, to the right: retraction), and curved orange arrows the moments in which the tongue changes direction in the reciprocating cycle. Light blue double-headed arrows show the dorso-ventral separation of the bill tips, and yellow double-headed arrows the dorso-ventral separation between maxilla and mandible at the bill base (both measured in lateral view; Fig. 4). (A) Tongue grooves collect nectar in a reservoir. (B) The retracting tongue makes contact with a closing bill and an intraoral meniscus starts to appear. (C) The bill tip closes, compressing the grooves and forcing nectar out of them, which is visualized as a front – proximal – meniscus of the liquid column. (D) The tongue is wrung out by protruding it through a small opening between the bill tips. (E) The tongue base splits the column of nectar inside the oral cavity by moving backwards and dragging the fluid with it. (F) This newly formed rear – distal – meniscus follows the tongue base closely upon retraction (Fig. S2).

In the dorsal-view backlit videos of *A. tzacatl*, we found that even near the end of tongue retraction a nectar meniscus moves backwards inside the bill (Figs 4 and 5A–C; Fig. S2), but that most of the internal bill space fills with liquid upon tongue protrusion (Fig. 5C,D; Movie 2). A potential explanation for the visible intraoral meniscus upon tongue retraction is that the nectar surrounding the tongue (Hainsworth, 1973) contacts the distal roof of the oral cavity and, as the tongue is being brought back with more liquid on top of it, the meniscus continues to move backwards. Then the bill tip closes rapidly (in about 20 ms; Figs 4 and 5B,C; Fig. S2), squeezing and preventing the escape of nectar, starting the wringing aided by the funnel-like internal space near the bill tip (Rico-Guevara and Rubega, 2017) and the tight match between tongue and intraoral capacities (Rico-Guevara, 2017). The tongue is pushed through the slit left by the bill tips being held nearly shut for a large proportion of the protrusion (Fig. 4; Fig. S2) and the nectar is forced out into the intraoral cavity as visualized in the inward meniscus displacement (Fig. 5C,D).

When the fluid is approaching the bill base, the meniscus at the front of the nectar column inside the bill becomes blurry (e.g. Movie 2). Ergo, the trace of the flow is truncated by the flow front ‘disappearing’ near the bill base; however, in some instances, a pause of the flow is detectable (e.g. when small bubbles are visible inside the nectar column), during which the tongue finishes its forward motion (Fig. S2). Once the tongue is maximally protruded and the bill is filled with nectar, the retraction of the tongue displaces the nectar column inside the bill backwards (Fig. 5E,F), and an irregular rear meniscus forms right in front – distal – of the tongue base (due to the splitting of the nectar column), suggesting that the tongue base is moving the liquid towards the throat (Fig. S2). In the dorsal view backlit high-speed videos, the tongue wings (as a V-shape) are visible as well as the distal meniscus that follows it upon retraction (Movie 2). However, a detailed frame by frame analysis was not possible even after monotonic enhancements (Movie 3), because both the V-shape and the meniscus blur out and only appear as diffuse shadows when a single image is in view (they only become apparent when several consecutive frames are viewed in quick succession). We suggest that future analyses could use more sophisticated backlit image enhancement (e.g. Buades et al., 2020) and tracking of fast actions (e.g. Garzón and Martínez, 2019).

The consistency of tongue and bill movements across species supports the idea that the mechanics of intraoral nectar flow are similar across species. The intraoral transport of a single nectar load is at least a two-step process; the first load stays inside the bill until the tongue base pushes it backwards, as a second load comes in (Fig. 5F), and so forth. A minimum of two licks is required to move a single nectar load from the flower to the throat (Fig. 5; Movie 3). This could explain why hummingbirds extend their tongues beyond their bill tips after foraging bouts (A.R-G., personal observation): they could be emptying the tongue grooves by extruding them completely and at the same time clear the inside of their bills by raking any remnants of nectar with their tongue wings. In order to maximize nectar uptake efficiency, offloading as much nectar as possible from the tongue is paramount for maintaining the maximum loading capacity across consecutive licks. After squeezing the tongue to unload the nectar inside the bill, the liquid must be transported towards the throat to be swallowed; if this process is not rapid and complete, the rate at which the bird can add additional nectar to the intraoral space is limited, and thus so is its overall intake rate. Hydraulic displacement could potentially move consecutively ‘stacked’ loads backwards, but we believe that when the tongue rakes a load mouthwards, the filling of an empty intraoral cavity would be faster than it would be if that load would need to push other ‘stacked’ loads on the way. We predict that for shorter protrusion distances, and/or longer bills, more than two licks would be required to transport a single load intraorally (see Discussion, ‘Eco-evolutionary implications’).

## DISCUSSION

### Comparisons with previous hypotheses

The first hypotheses on how nectar is moved through the bill to be swallowed date back to the 19th century, with renewed attention in the 1980s and within the last 20 years (reviewed in Rico-Guevara, 2014; Cuban et al., 2022). There seem to be a variety of potential solutions to the fluid dynamics challenge of efficiently and delicately extracting minute amounts of nectar, which often requires thin and elongated beaks and protrusible tongues to reach inside long and tubular flowers (Hewes et al., 2022). A narrow bill as a special tool to navigate corollas presents the additional challenge of moving the liquid to the, in most species, distant throat. Now, we finally have experimental data for a missing piece of a long-standing feeding mechanics puzzle: how the nectar is freed from the tongue (after it is collected in the flower and brought inside the bill) and transported to the pharynx (Cuban et al., 2022). As we were able to visualize menisci inside the bill, we could thus distinguish filled and empty spaces inside the oral cavity throughout the lick cycle. We summarize the expeditiously coordinated (to match the high licking rate) and complementary intraoral transport mechanisms as follows. (1) Distal wringing: the tongue starts to be unloaded as soon as it is retracted, and the nectar is completely wrung out of it upon protrusion; this unloading occurs near the bill tip where the distal intraoral capacity is decreased when the bill tips are closed. (2) Tongue raking: the nectar filling the intraoral cavity is retrieved by the tongue base, leveraging flexible flaps to act as a dynamic valve, upon retraction. (3) Basal expansion: when more nectar is being released inside the oral cavity with the filling front moving proximally, the jaws separate basally (phase-shifted from the bill tip opening), increasing the intraoral capacity at the base, thus facilitating the reception of the nectar aliquot to be swallowed. Expanded explanations of these mechanisms are found in the Supplementary Materials and Methods (see ‘Detailed mechanistic hypotheses’). From the five nectar-feeding specific hypotheses noted in the Introduction, our findings are only compatible with hydraulic displacement (wringing) and cohesive suction (base expansion). Cohesive pulling seems to only drag a very small amount of nectar backwards as a layer in front of the tongue base, and almost all of the fluid is instead pushed (raked) backwards.

**Fig. 6.**
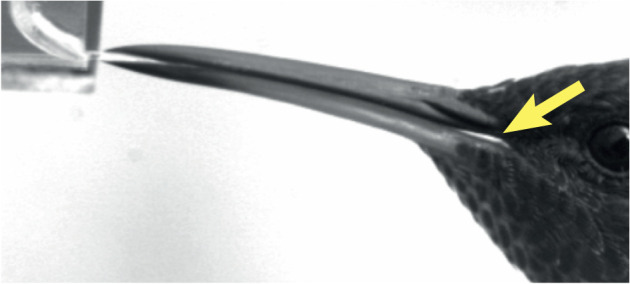
**Lack of a seal at the bill base does not preclude nectar drinking.** Still frame from a high-speed video of an indigo-capped hummingbird (*Saucerottia cyanifrons*) drinking nectar from an artificial feeder. Note that there is an opening (light) at the base of the bill (yellow arrow).

When considering fluid motion through a narrow channel (such as the intraoral space in elongated bills), spontaneous capillary flow with a meniscus pulling the liquid column is expected (reviewed in Kolliopoulos and Kumar, 2021). For capillarity to work, the bill base cannot be hermetically closed and a concave front meniscus would be visible. Both of those requirements are compatible with our observations (e.g. Fig. 6; Movie 2); however, for capillarity to be the main driving mechanism, a longer time span (with a pause of a fraction of a second) for the meniscus to pull the column, and fill the intraoral cavity, would be necessary. Intraoral transport in hummingbirds must be coordinated with tongue reciprocation at rates up to 20 times per second (with no pauses along the lick cycle) and capillarity equations on narrow tubes indicate that resulting menisci speeds are too slow to keep up with the fast pace of the tongue (Rico-Guevara et al., 2015). The above calculations have been performed for groove dimensions about a quarter of the diameter of the intraoral cavity (Rico-Guevara, 2017), and capillary flow is slower in larger tubes (Hamraoui et al., 2000; Baek et al., 2021). Furthermore, in initial licks of each drinking bout – where the intraoral cavity is not coated with nectar – the intraoral meniscus is not concave as expected under the capillarity pulling mechanism (the flat meniscus observed suggests that the fluid is being pushed instead; e.g. first lick in Movie 2). Lastly, capillarity would be able to pull nectar through the bill while there is a connection to a reservoir and the tongue is used as a bridge. Once the tongue is outside the nectar and an aliquot is trapped in the grooves, given that the edge contact length (where adhesion between the liquid and the inner walls occurs) between the liquid column and the tube is proportional to the radius of the tube (Kolliopoulos and Kumar, 2021), capillary pulling alone would not be able to pull nectar out of the grooves because they have a much smaller diameter than the intraoral cavity (and their internal surface is not hydrophobic; Rico-Guevara, 2017).

Observations of gaps at the bill base (e.g. Fig. 6) oppose hypotheses involving the generation of a vacuum at the bill base or at the throat region (Döhling, 1931; Steinbacher, 1935), or hermetic suction. Our observations of continuous tongue reciprocation along with its interaction with the fluid do not support the hypothesis of suction through a vacuum between the tongue and the palate near the bill base (Gadow, 1883). We did not find evidence for ‘cohesive pulling via tongue retraction’ (Moller, 1930; Scharnke, 1931), a process analogous to a front-acting reciprocating pump mechanism (e.g. syringes, where the liquid stays on the opposite side of the plunger support), in which the barrel is the bill and the plunger is the tongue, so that the liquid column is pulled in front of the seal of the plunger. We found that the tongue (plunger) is pulled too fast to prevent rupturing of the nectar column, and thus only a small meniscus follows in front – distal – of the tongue base after the column splits (Fig. 5; Movies 2 and 3). Therefore, most of the fluid is moved proximally on the backside of the tongue base, i.e. the nectar is not being pulled in front but it is being pushed behind the tongue wings, like a rake collecting leaves. A rake applies force behind the object (in terms of its path), so we propose a mechanism in which the tongue base/wings function as a back-acting reciprocating pump with a dual plate check valve for a piston (see Supplementary Materials and Methods). We did not find evidence of extensive lateral mandibular expansion at the bill base (Yanega and Rubega, 2004; Zusi, 2013) during drinking, but we did find basal dorso-ventral separation between the mandible and maxilla that potentially could create ‘cohesive suction through bill base expansion’ (*sensu* Cuban et al., 2022). Combining this with the support we found for ‘hydraulic displacement via tongue wringing’ (Ewald and Williams, 1982; Rico-Guevara and Rubega, 2017; Cuban et al., 2022) also suggests that the phase shift between compression and expansion of proximal and distal intraoral cavity could aid the flow in a similar way in which the phase-shifted cibarial and pharyngeal pumps do in mosquitoes (e.g. Kikuchi et al., 2018). The phase offset values vary significantly between the videos of individuals analysed (Table S2), with large standard deviations per species (Table S3), potentially suggesting that it is dependent on changes in the lick cycle (e.g. tongue protrusion). This phenomenon warrants further study to determine what internal or external factors are influencing phase-shifted bill movements during feeding. 3D fluid dynamics modelling considering the internal volumes of the structures involved (e.g. using CT scans; Rico-Guevara, 2017), would be able to test whether the hypothesized forces we describe in this paper can account for the intraoral transport of the nectar.

### Eco-evolutionary implications

Understanding the intraoral transport mechanism opens the doors to explore its influence on ecological and evolutionary trends; for instance, the potential cost of having elongated bills in terms of the time and energy invested in moving fluid through a longer tube. A recent review discusses mechanistic and behavioural (e.g. agonistic interactions) rationales of the selective forces likely to be involved in bill length/shape evolution (Rico-Guevara et al., 2021); here, we provide brief considerations on this topic, particularly in relation to intraoral transport among species of different bill lengths (e.g. Fig. 3; Table S1). Longer bills could be beneficial to hummingbirds by having larger internal capacities and accommodating longer tongues that can collect more nectar per lick (e.g. Hainsworth, 1973), as well as facilitating probing inside long corollas (Snow and Snow, 1972; Stiles, 1975; Wolf et al., 1972). Inserting the bill farther results in the tongue bridging a shorter gap between the nectar at the bottom of the flower and the bill tips, permitting higher licking rates (Ewald and Williams, 1982) and wringing nectar loads off the tongue more rapidly. We hypothesize that working through hydraulic displacement (Cuban et al., 2022), several aliquots would be accumulated in quicker succession, with each one pushing the previous one further inside the bill.

Bills of hummingbirds tend to be similar in length and curvature to the corollas of the flowers they usually feed on (Snow and Snow, 1972, 1980; Stiles, 1975; Wolf et al., 1972, 1976; Gutiérrez-Zamora, 2008; Maglianesi et al., 2014, 2022; reviewed in Rico-Guevara et al., 2021). As foraging efficiency (caloric value obtained relative to the caloric costs for obtaining food) can act as a selective pressure (Schoener, 1971; Tullock, 1971; Houston and McNamara, 2014), individuals of a given hummingbird species (or sex) would be expected to be more efficient (minimizing handling time) while feeding on flowers matching their bill length and shape (Wolf et al., 1972; Temeles and Roberts, 1993; Temeles et al., 2000, 2010). However, tests of this ‘bill–corolla matching increasing efficiency’ hypothesis have produced conflicting results; it would be expected that long-billed species would be more efficient with long flowers and short-billed hummingbirds would be more efficient with short flowers, but in fact previous research has failed to support the second prediction (Hainsworth, 1973; Montgomerie, 1984; Temeles and Roberts, 1993; Temeles, 1996; Temeles et al., 2002, 2009). Specifically, experiments have found that longer-billed birds feed faster from longer flowers than shorter-billed birds, but shorter-billed birds do not feed faster from shorter flowers than longer-billed ones (reviewed in Temeles, 1996). Given that regardless of corolla length, longer bills seem to be able to achieve smaller distances between the bill tip and the nectar than shorter bills, achieving elevated nectar extraction efficiency (Hainsworth, 1973; Hainsworth and Wolf, 1976; Montgomerie, 1984; Grant and Temeles, 1992; Temeles and Roberts, 1993; Temeles, 1996), it may seem that evolving a longer bill will be a functionally selected adaptive peak. Longer-billed hummingbirds could potentially access a wider floral spectrum; however, they may not be able to find enough nectar on small flowers (Snow and Snow, 1980) because they are preferentially visited by short-billed hummingbirds (unless they nectar rob). Therefore, the recurrent pattern of bill–corolla matching (e.g. Weinstein and Graham, 2017; Dalsgaard et al., 2021) would be the result of interspecific and intersexual (e.g. Maglianesi et al., 2022) competition among coexisting species with different bill lengths and shapes. Nonetheless, the generalized bill–corolla matching pattern still does not explain the evolutionary persistence of short-billed hummingbirds, but it does point to the existence of drawbacks associated with long bills, otherwise short bills would ultimately be outcompeted.

Proposed disadvantages of possessing a longer bill include making more insertion errors when feeding in narrow flowers (compared with shorter-billed hummingbirds; Temeles, 1996) rendering diminished net energy gain per visit, and complicating adjustment of the bill tip inside the flower to access the nectar chamber (Rico-Guevara et al., 2021). Along similar lines, short-billed hummingbirds would be able to more easily cling when nectar feeding (or land while drinking from flowers on the ground), simply because their bodies will be closer to substrates they could grasp. Energy savings from decreased hovering expenditure would also increase net energy gain. In addition to the abovementioned ‘mechanoethological’ (Green et al., 2021) drawbacks of having longer bills, based on the work we present here, we hypothesize that longer-billed species may experience longer intraoral transport times as a result of the liquid traversing larger distances. Although this hypothesis is not supported by the experimental evidence that shorter-billed birds do not feed faster from shorter flowers than longer-billed ones (e.g. Temeles, 1996), several aspects need to be taken into consideration. First, the effects of longer intraoral transport distances on handling time would probably be noticeable among species with large differences in bill lengths (unlike previous experiments; reviewed by Temeles, 1996). Second, measurements on a single foraging bout (or sequential bouts separated by some travel time, e.g. Montgomerie, 1984) may not reflect this increased transport time as long-billed hummingbirds could leave the flower with their bills full and finish the intraoral transport after the time counter (e.g. stopwatch) in the experiment had been stopped. The ecological advantage of quicker intraoral transport times for short-billed species would manifest while hummingbirds are visiting many flowers in rapid succession, such as helmetcrests (genus *Oxypogon*) feeding in short corollas packed tight in inflorescences (e.g. in the family Asteraceae) that allow quick bill transitions. Third, under experimental conditions, if equal access to the nectar chamber is provided, an increased intraoral transport time may be compensated for by increased intralingual (e.g. Hainsworth, 1973) and intraoral capacity, however, it is likely that smaller bills exhibit thinner bill tips and tongues (with associated smaller bill tip separations; Fig. 3) able to better navigate the reduced spaces inside smaller flowers. Experiments controlling for, and studies taking into consideration, not only corolla diameter (Temeles, 1996; Temeles et al., 2002) but also the internal geometry of the floral tube and access to the nectar chamber are warranted (Grant and Temeles, 1992; Rico-Guevara et al., 2021). Fourth, the advantage of longer-billed hummingbirds in maintaining close bill tip–nectar distances on the one hand is beneficial as it permits higher licking rate but on the other implies a shorter intraoral distal reach of the tongue base. Shorter-billed hummingbirds are expected to have faster intraoral transport times as they can empty a larger proportion of their intraoral capacity with the tongue base at every lick. This suggests that longer-billed species would rely more heavily on out of phase base–tip separation (Figs 1 and 2) and successive wringing (Cuban et al., 2022), requiring more licks to move a load of nectar (maintaining a given tongue protrusion) than shorter-billed hummingbirds. Supporting this idea, the species with the longest bill in our dataset exhibits the absolute and proportionally largest bill base separation, indicating a heightened importance of this mechanism for intraoral transport (*P. longirostris*; Fig. 3; Fig. S1). And fifth, given that distally, hummingbird tongues are inert structures without known mechanoreceptors (Rico-Guevara, 2017), the sensory feedback regarding the presence or depletion of nectar in a flower would be delayed in longer-billed species if they indeed have longer intraoral transport times (more lick cycles needed to tell whether/when the nectar chamber is empty, e.g. fig. 4 in Rico-Guevara et al., 2021), which will further increase handling time and reduce foraging efficiency.

With the caveat that all of the aspects mentioned above are inferences and experimental data are required to test each one of the aforementioned hypotheses, from a biomechanical point of view, bills should only be as long as absolutely needed. The existence of many hummingbird species with exceptionally short bills could be a clue to the importance of intraoral transport efficiency and its effect in bill length evolution. Our study helps to unveil more specific hypotheses and predictions on how bill length and shape affect and are evolutionarily constrained by the intraoral transport mechanisms. We suggest that future studies focus specifically on the effect of bill curvature on hummingbird oral transport mechanics, which could be an additional factor accounting for the differences in jaw separation among species (Figs 2 and 3; Fig. S1). Although beyond of the scope of this paper, we underscore that curvature interacts in complex ways with length in the co-evolutionary process resulting in bill–corolla matching (reviewed by Rico-Guevara et al., 2021). An in-depth comparison of drinking efficiency – simultaneously measuring tongue protrusion and immersion distances – across the wide morphological medley of hummingbirds is sorely needed.

The discoveries from the work detailed in this paper reveal a crucial component of nectar feeding in hummingbirds that, although as relevant as nectar collection by the tongue (summarized in Rico-Guevara et al., 2019a), had previously been unstudied. A full grasp of every stage of the drinking process is the necessary first step to enable predictive modelling in terms of drinking efficiency of a given bill under determined floral access and nectar properties. This approach could link the vast hummingbird morphological variation to differential performance on the wide variety of bird bill–flower corolla pairings (Rico-Guevara et al., 2021). With this first experimental exploration of intraoral transport of nectar, the foundation is set for comparative biomechanical studies to evaluate the diverse nectar feeding hypotheses (Cuban et al., 2022) and associated adaptations of unrelated clades of nectarivorous taxa (e.g. honeyeaters, sunbirds and many others; Hewes et al., 2022), to better elucidate the extent of convergent and alternative solutions to this unique feeding challenge. Our findings highlight the importance of studying the morphofunctional adaptations of not only the tip but also the base of the tongue of nectarivorous taxa. Our research portrays tongues as dynamic structures that can only be understood in action, rather than inferring their functioning based only on their morphology. Similarly, expanding the study of nectar drinking beyond the liquid collection stage would provide cues to the limits of consumption capabilities, and of the evolutionary interplay between the loading and offloading mechanisms. For example, nectarivores (such as birds and bats) that drink nectar with their mouths open would be prevented from using distal squeezing (e.g. wringing through the bill tips) to transfer liquid from the tongue to the mouth, which will impose constraints on the subsequent intraoral transport mechanisms. Understanding this linkage between morphology and performance is vital to fully understand nectarivores' foraging decisions and competitive strategies (e.g. Sargent et al., 2021), with ultimate implications for their co-evolution with the plants they pollinate.

## Supplementary Material

10.1242/jexbio.245074_sup1 Supplementary information
