# Supplementary material for: Nectar feeding beyond the tongue: hummingbirds drink using phase-shifted bill opening, flexible tongue flaps and wringing at the tips
Source: J Exp Biol. 2023 Apr 3;226(Suppl 1):jeb245074. doi: 10.1242/jeb.245074 (PMC10112966; doi:10.1242/jeb.245074)
Supplement: Supplementary information [file jexbio-226-245074-s1.pdf]

Journal of Experimental Biology • Supplementary information

Supplementary Materials and Methods

Bill movements phase shift measurements

Videos of hummingbirds drinking nectar from artificial feeders were digitized using DLTDV8 (Hedrick, 2008) to mark points of interest in each frame. The upper bill tip, lower bill tip, upper bill base, and lower bill base were digitized for enough frames to encompass 10 complete licks without any obstructions from the corolla, wings, or the bird moving its bill base out of frame. The bill tips were defined as the most distal portion of the keratinized bill for both the maxillary and mandibular rhamphothecae. The lower bill base point was the point on the lower bill that is just past the distal most point of soft tissue of the throat (to avoid digitizing the throat feathers/skin) and the upper bill base point was defined as the point on the upper bill in the same transverse plane of the bill as the lower bill base point. Both of these points tended to coincide within the same transverse plane as the distal point of the nares. Using a MATLAB script, we calculated the distance between the bill tip points and the distance between the bill base points for all frames of video during which we digitized the licking. Both sets of distances oscillated at the same frequency as the licking cycle (one opening and closing of the bill tip and bill base per protraction and retraction of the tongue) and we calculated the phase offset between the two sets of distances as they oscillated (Table S2). This resulted in a single value in degrees of offset from 0 to 360 degrees with 180 degrees meaning that the bill tips would be open while the bill base closed and vice versa, while 0 or 360 degrees meaning that the bill tip and bill base are open or closed at the same time. We collected bill tip and base digitization data for 24 individuals of seven species of hummingbirds (Table S3).

External measurements

For one individual of the seven species studied, we collected complete bill profile digitization data for 10 complete licks. A complete lick sequence was defined as the time from when the bill begins to open (start of the tongue protrusion), through the extension and immersion of the tongue tip in the nectar, and until the tongue is completely retracted and the bill completely closes again. For all lick sequences (which were of different durations), we extracted a subset of 11 equally spaced frames to analyse in the lateral and dorsal views (yielding 10 time steps). The starting frame was selected as the one immediately preceding the first visualization of the tongue outside the bill, and the last frame was the corresponding one for the next lick, thus completing a full cycle. For each of the 11 frames per lick, we digitally traced two bill contours, to create upper and lower bill profile lines, using tpsDIG2 (Rohlf, 2010). The first line followed the culmen beginning distally at the maxillary tip and ending at the most proximal point of the exposed culmen (the point at which the feathers start, Fig. 1A). A line perpendicular to the bill axis was followed down from the exposed culmen's most proximal point (culmen base), and its intersection with the ventral bill contour was used to place a point defining the most proximal point of the ventral bill profile. We traced the profile of the mandibular ventrum (defined here as the lower jaw contour, the ventral counterpart of the exposed culmen) from the most proximal point as defined above to the mandibular tip (Fig. 1A). The bill profile lines were then resampled using tpsDIG2 so that each line had 21 equidistant semi-landmarks, respectively. All corresponding points along the bill profiles were used to assign bill regions, beginning at the bill base (region 1) and ending at the bill tip (region 20), yielding 5% increment length steps along the bill (Fig. 1A).

Detailed mechanistic hypotheses of intraoral nectar transport

Putting together the external measurements for all of the species and the intraoral observations for *Amazilia tzacatl*, we can encapsulate the nectar transport inside the bill into three distinct mechanisms:

(1) Distal wringing: The maximum separation at the bill base occurred at a time between 30% and 50% of the lick cycle duration (during tongue protrusion), and the base minimum separation between 70% and the 90% of the lick cycle (during tongue retraction, Figs. 1B, 2, 4, S2). Conversely, the bill tip maximum separation occurred

between 50% and 70% of the cycle (shortly after the tongue reaches maximum protrusion and starts retraction), and all the licks were synchronized using the bill tip minimum separation, at the start and end of each lick sequence (Fig. 2). While the tongue is being retracted the bill tips are kept far enough apart (the aperture is just big enough) to allow the tongue loaded with nectar to enter the bill. Then, upon protrusion the tongue grooves are squeezed through a small aperture at the bill tips for as long as they can be extruded (Figs. 1B, 2, S2, Rico-Guevara and Rubega, 2017). We do not present measurements of tongue compression, given that a thorough treatment of the differences in dorso-ventral tongue thickness, confirming that the grooves are actually compressed has already been published (Rico-Guevara *et al.*, 2015).

(2) Tongue raking: We find the bill tubular geometry and tongue reciprocating movement to resemble a positive-displacement pump (*e.g.*, manually operated, Marshall, 2017). Under this scenario, the tongue base is displacing the liquid column only during its retraction, actively "raking" it proximally (Fig. 5, S2, Movie S3). We hypothesise that the tongue base behaves as a dynamic one-way valve, with the flexible tongue wings acting analogously to the semi-discs of a dual plate check valve (Sotoodeh, 2018), with the difference that the valve is not static in space, but it moves across the intraoral space (as in a manual well pump). The effectiveness of this one-way valve at the tongue base would be facilitated by coordinated shifts in internal oral capacity, the phase-shift between proximal and distal portions, and nectar flow (Movie S3). The reduction of the volume of the distal oral cavity, and the tongue protrusion through a small aperture, extrude the nectar from the tongue hydraulically pushing it proximally at the same time when the valve is moving distally (Movie S3). When the tongue base is moving towards the tip, this 'flexible seal' would passively compress radially allowing nectar to flow around it (the tongue wings would fold against the tongue body as the semi-discs of a check valve). The nectar flow inside the bill forced from the distal compression stops when the tongue has been fully extruded, by then a liquid column has entirely filled the oral cavity and the tongue base is at the distal end of it (Fig. 5, S2). Then, as the plunger is retracted the seal passively expands out again (tongue wings unfold as the semi-discs forming a flat-disc seal) allowing the tongue base to rake the nectar proximally (Fig. S2, Movie S3). A lack of a hermetic seal near the base (*e.g.*, Fig. 6) allows air to escape near the bill base in front of the proximally moving nectar front (which could also occur through the choana –passage in the palate connecting oral and nasal cavities – letting air out through the nares). At this point the jaws are separating at the bill base increasing the basal oral cavity volume and he aliquot of nectar is swallowed.

(3) Basal expansion: To assess how the bill movements are coupled with the tongue motion, we time-matched measurements of the bill base and tips separation with those of the intraoral displacement of the tongue base and the external kinematic state of the tongue tip (Figs. 1B, 4, S2). The effect of the phase shift between the opening and closing of the basal and distal regions maximizes at a point around 70% of the licking cycle for most species (Figs. 1B, 2, 5, S1). Just before the tongue base is the closest to the bill tip (maximum protrusion) the bill base achieves maximum separation; the internal basal intraoral volume is the largest right before the tongue base is the farthest from the bill base (Figs. 4, S2). Proximal displacement of the nectar column by the tongue base is accompanied by a dorso-ventral expansion of the bill base, and filling of the bill with nectar is coincident with a reduction of the separation between the bill tips. The change in internal cross-sectional area of the bill (*e.g.*, Fig. 2 in Rico-Guevara, 2017) is phase-shifted along the length of the bill throughout the feeding cycle (Figs. 1, 2, 4, S2), which we hypothesise leads to changes in flow resistance that facilitate intraoral flow.

The base-tip opening phase shift leaves a middle zone of least dorso-ventral separation, which varies across species but it is never at the bill tip or bill base. Functionally, it could be a necessary hinge zone to allow for the tip-base out-of-phase motions, but also it could serve as a constriction that facilitates the retrieval of the nectar load by the tongue base (making a tighter match between the tongue wings and the roof of the intraoral cavity), and it could also speed up the flow through the middle of bill by maintaining a smaller cross sectional area (allowing for the increased capacity of the bill base opening to aid nectar flow to the throat).

Literature cited

Hedrick, T. L. (2008). Software techniques for two- and three-dimensional kinematic measurements of biological and biomimetic systems. *Bioinspir. Biomim.* **3**: 034001.

Marshall, K. C. (2017). An evaluation of the water lifting limit of a manually operated suction pump: Model estimation and laboratory assessment (10639461). Available from ProQuest Dissertations & Theses Global.

Rico-Guevara, A., Fan, T-H., and M. A. Rubega. (2015) Hummingbird tongues are elastic micropumps. *Proc Biol Sci* **282**:20151014

Rico-Guevara, A., and Rubega, M. A. (2017). Functional morphology of hummingbird bill tips: their function as tongue wringers. *Zoology* **123**: 1-10.

Rico-Guevara, A., and Rubega, M. A. (2017). Functional morphology of hummingbird bill tips: their function as tongue wringers. *Zoology* **123**: 1-10.

Rohlf, F. J. (2010) *TPSDig2 Version 2.16* (Department of Ecology and Evolution, Stony Brook University, New York).

Sotoodeh, K. (2018). Comparing dual plate and swing check valves and the importance of minimum flow for dual plate check valves. *American Journal of Industrial Engineering* **5**: 31-35.

Journal of Experimental Biology • Supplementary information

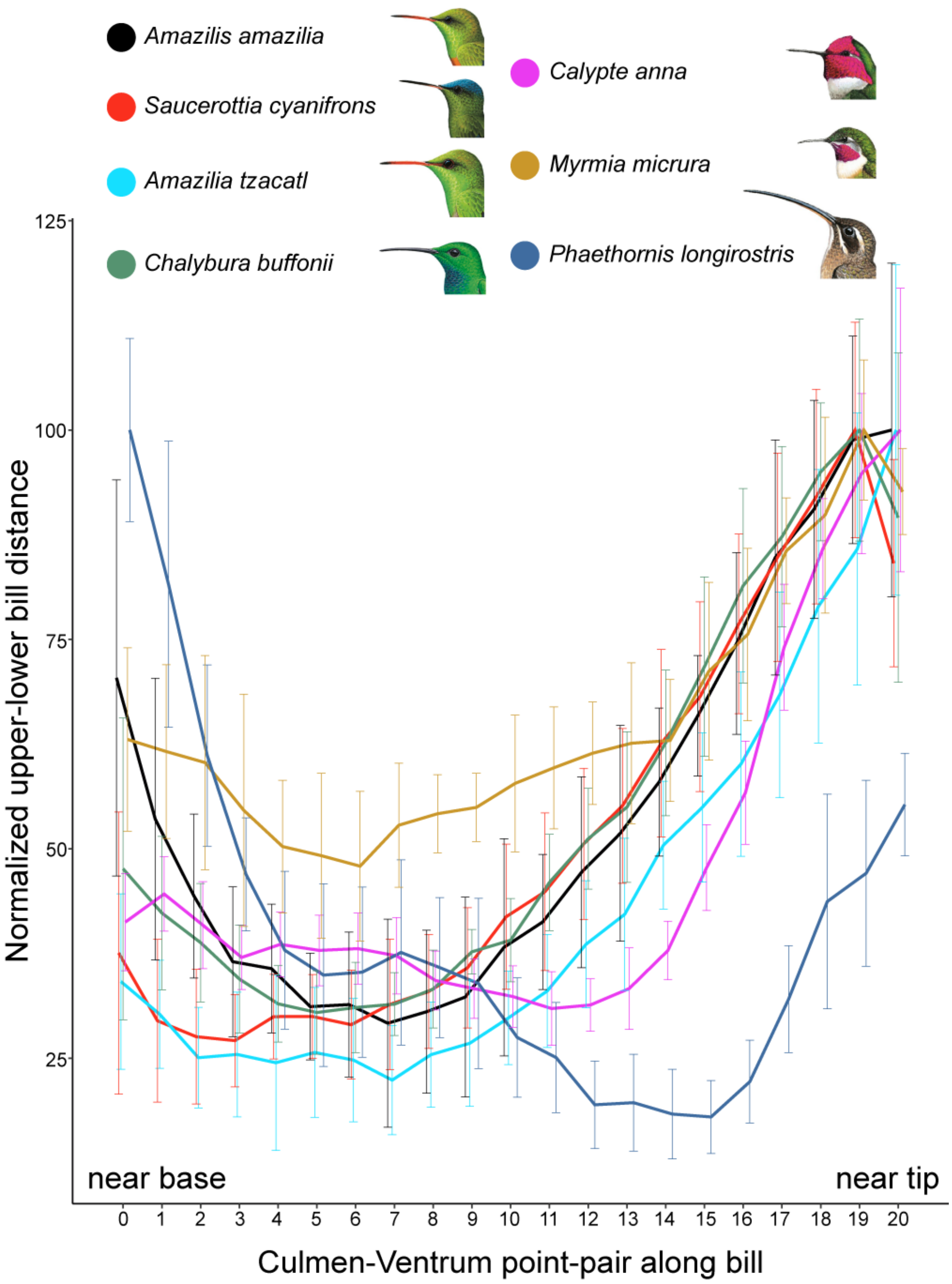

Fig. S1. Ranges of separation (proportional to maximum point-pair range per species) among culmen and mandibular ventrum point-pairs for seven species of hummingbirds. We obtained the range per point-pair by subtracting the maximum and minimum separation values along the licking cycle. We used ten randomly selected licks from different foraging bouts and reported average patterns (with corresponding standard deviations) per species. Head profiles from Birds of the World | Cornell Lab of Ornithology. We provide statistical analyses testing the differences among these curves (Figs. S3, S4).

Journal of Experimental Biology • Supplementary information

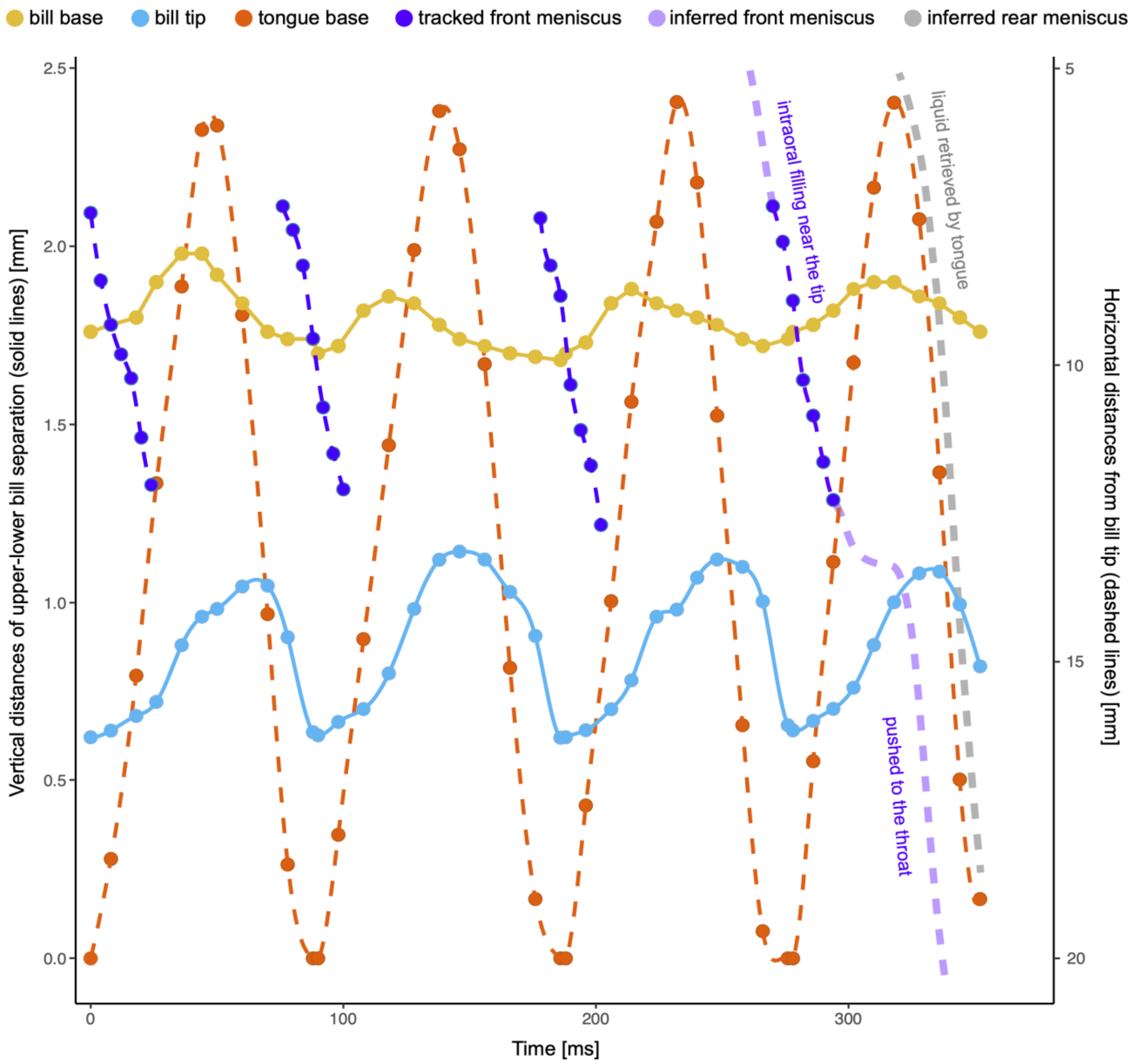

Fig. S2. Bill and tongue movements in relation to intraoral flow for four consecutive licks of a Rufous-tailed Hummingbird (*Amazilia tzacatl*). The *X-axis* displays the continuous time in milliseconds. The *left Y-axis* shows the dorso-ventral separation in millimetres (mm) at the bill base (yellow solid line) and at the bill tip (light blue solid line). The *right Y-axis* portrays distances from the bill tip towards the bill base (in mm). Inferred motion of the tongue base (red dashed line) varies from near 5 (maximum protrusion) to near 20 (tongue entirely inside the bill). Intraoral flow of nectar (indigo dashed line), measured as the distance between the proximal nectar meniscus and the bill tip, was only trackable behind the black distal region (about 7 mm from the tip, Fig. 3); the discontinuity is caused by the proximal meniscus of one load of nectar

Journal of Experimental Biology • Supplementary information

moving outside of the camera frame and into back of mouth/throat. In some cases, we could visualize (but not track due to blur) the front meniscus further inside the bill, where there was a pause (the liquid stopped advancing) that lasted until the tongue started to move backwards (lavender dashed line). At this moment, the nectar column inside the oral cavity splits, coinciding with the backward movement of the tongue based and a rear meniscus (too blurry to be tracked, grey dashed line) follows the tongue base closely upon retraction (Fig. 5, Movies S2-S4). Data for the interpolated lines was generated using a cubic-spline script in MATLAB with each point being 1 ms apart.

A

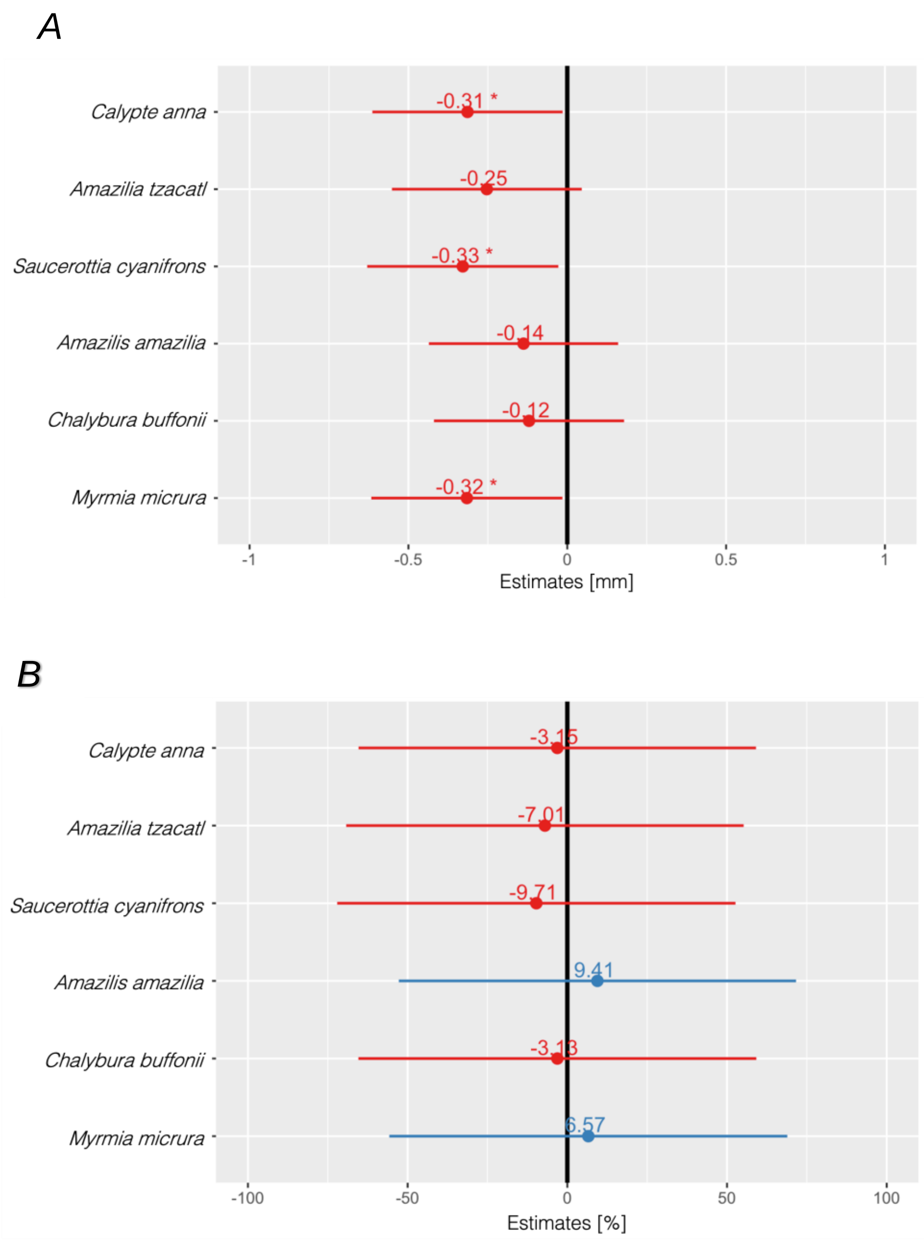

Fig. S3. Forest plots showing interspecific differences of bill separation comparing all of the species to the one with the longest bill (*Phaethornis longirostris*). *(A) Absolute values:* The range of separation curves using the differences in millimetres (X-axis) between upper and lower jaws (Fig. 3) were analysed via GLMMs with 1D spatial autocorrelation. Where the confidence interval excludes zero, we might infer significant differences between that species and the Long-billed Hermit (see methods). The three species with shortest beaks (Table S1) show significant differences. *(B) Normalised values:* The range of separation curves normalising the differences in millimetres to maximum separation of each species (% in the X-axis) between upper and lower jaws (Fig. S1) were analysed via GLMMs with 1D spatial autocorrelation. Since none of the confidence intervals excludes zero, we infer that there are no significant differences between the species (see methods).

Journal of Experimental Biology • Supplementary information

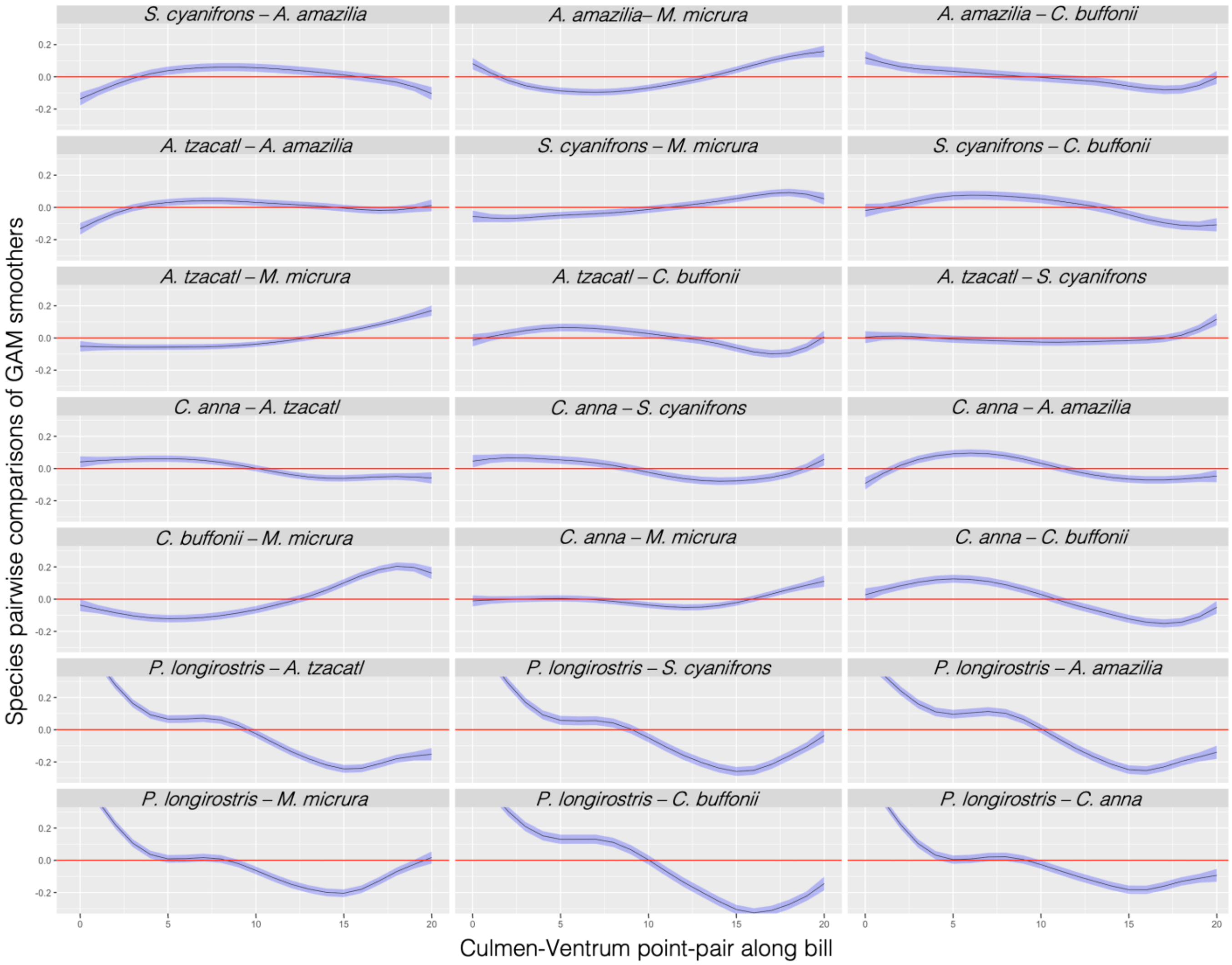

Fig. S4. Interspecific comparisons between the ranges of separation curves along the bill length. All of the 21 pairwise comparisons among the seven species are presented. The X-axes, as in Figs. 3 and S1 represent the position along the bill length; the bill base is on the left (point 0) and the bill tip is on the right (point 20). The curves represent the differences between 'GAM smoothers' (see methods), where the confidence interval excludes zero (the blue shade does not overlap with the red line), we might infer significant differences between a pair of estimated smooths at a given point along the bill.

Table S1. Hummingbird species included in the bill motion analyses. We organize the species taxonomically to highlight the taxonomic breath of the sampling, and order them according to bill length. We also add place of filming and sex of the individuals for which detailed graphs were generated (presented in the main text).

**Clade**	**Genus**	**species**	**Bill length (mm)**	**Country**	**Sex**
Hermits	*Phaethornis*	*longirostris*	40.2	Ecuador	Female
Emeralds	*Chalybura*	*buffonii*	23.3	Colombia	Male
	Amazilia	*tzacatl*	20.2	Ecuador	Male
	Amazilis	*amazilia*	19.4	Colombia	Female
	Saucerottia	*cyanifrons*	16.8	Colombia	Male
Bees	*Calypte*	*anna*	16.3	USA	Male
	Myrmia	*micrura*	15.7	Ecuador	Female

Journal of Experimental Biology • Supplementary information

Table S2. Bill tip and bill base separation phase offset values per individual. We tracked the upper bill tip, lower bill tip, upper bill base, and lower bill base to measure the separation the bill tips and of the bill base. The degree of phase offset was measured as the difference between the time of maximum separation of the tips and the time of maximum separation of the tips, which to compare across species is transformed to degrees (360° equals the time of a full cycle of opening and closing of the bill tip and base), 0° or 360° means no offset, the tip and base are open or closed at the same time. The maximum offset possible is 180°, where the bill tip would be completely open while the bill base is completely closed or vice versa. Individuals marked with * are the ones for which details graphs are presented in the main text, and their bill lengths presented in Table S1.

**Individual**	**Degree_Phase_Shift (0 to 360°)**
*Phaethornis_longirostris_1**	273.0798719
Phaethornis_longirostris_2	235.6100753
Phaethornis_longirostris_3	115.5142165
Phaethornis_longirostris_4	110.8654016
*Chalybura_buffonii_1**	308.2020758
Chalybura_buffonii_2	188.404308
Chalybura_buffonii_3	61.29293211
Chalybura_buffonii_4	122.9225379
*Amazilia_tzacatl_1**	265.2230791
*Amazilis_amazilia_1**	270.0290742
Amazilis_amazilia_2	171.0694395
Amazilis_amazilia_3	250.8103849
Amazilis_amazilia_4	51.40167212
*Saucerottia_cyanifrons_1**	276.8775805
Saucerottia_cyanifrons_2	65.13545512
Saucerottia_cyanifrons_3	337.7329228
Saucerottia_cyanifrons_4	339.4972271
*Calypte_anna_1**	238.4493575
Calypte_anna_2	72.5713314
Calypte_anna_3	86.24314445
*Myrmia_micrura_1**	305.7099555
Myrmia_micrura_2	47.19553905
Myrmia_micrura_3	87.91086772
Myrmia_micrura_4	138.2614635

Journal of Experimental Biology • Supplementary information

Table S3. Bill tip and bill base separation phase offset values per species. We tracked the upper bill tip, lower bill tip, upper bill base, and lower bill base to measure the separation the bill tips and of the bill base. The degree of phase offset was measured as the difference between the time of maximum separation of the tips and the time of maximum separation of the tips, which to compare across species is transformed to degrees (360° equals the time of a lick cycle), 0° or 360° means no offset, the tip and base are open or closed at the same time. The maximum offset possible is 180°, where the bill tip would be completely open while the bill base is completely closed or vice versa. Values per individual are presented in Table S2.

**Species**	**Average Degree_Phase_Shift (0 to 360°)**	**SD Degree_Phase_Shift (0 to 360°)**	**# of individuals per species**
Phaethornis_longirostris	183.7673913	82.94090074	4
Chalybura_buffonii	170.2054635	105.6280857	4
Amazilia_tzacatl	265.2230791	NA	1
Amazilis_amazilia	185.8276427	99.33241159	4
Saucerottia_cyanifrons	254.8107964	129.7581708	4
Calypte_anna	132.4212778	92.07711379	3
Myrmia_micrura	144.7694564	113.5748896	4

Journal of Experimental Biology • Supplementary information

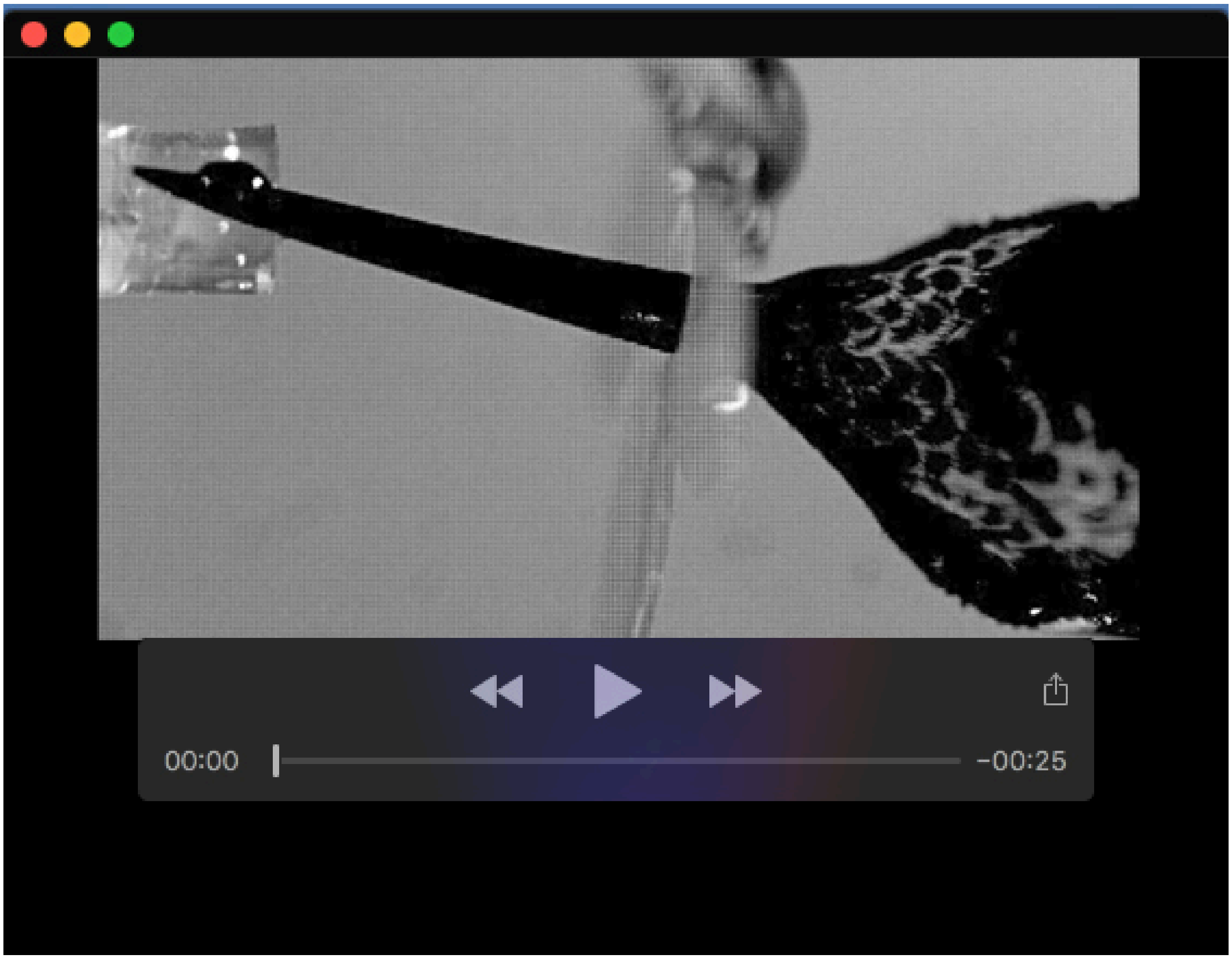

Movie 1. Ventral view of a hummingbird drinking nectar. High-speed video (1000 fps) of an Anna's Hummingbird (*Calypte anna*) in which no extensive lateral separation of the rami is visible.

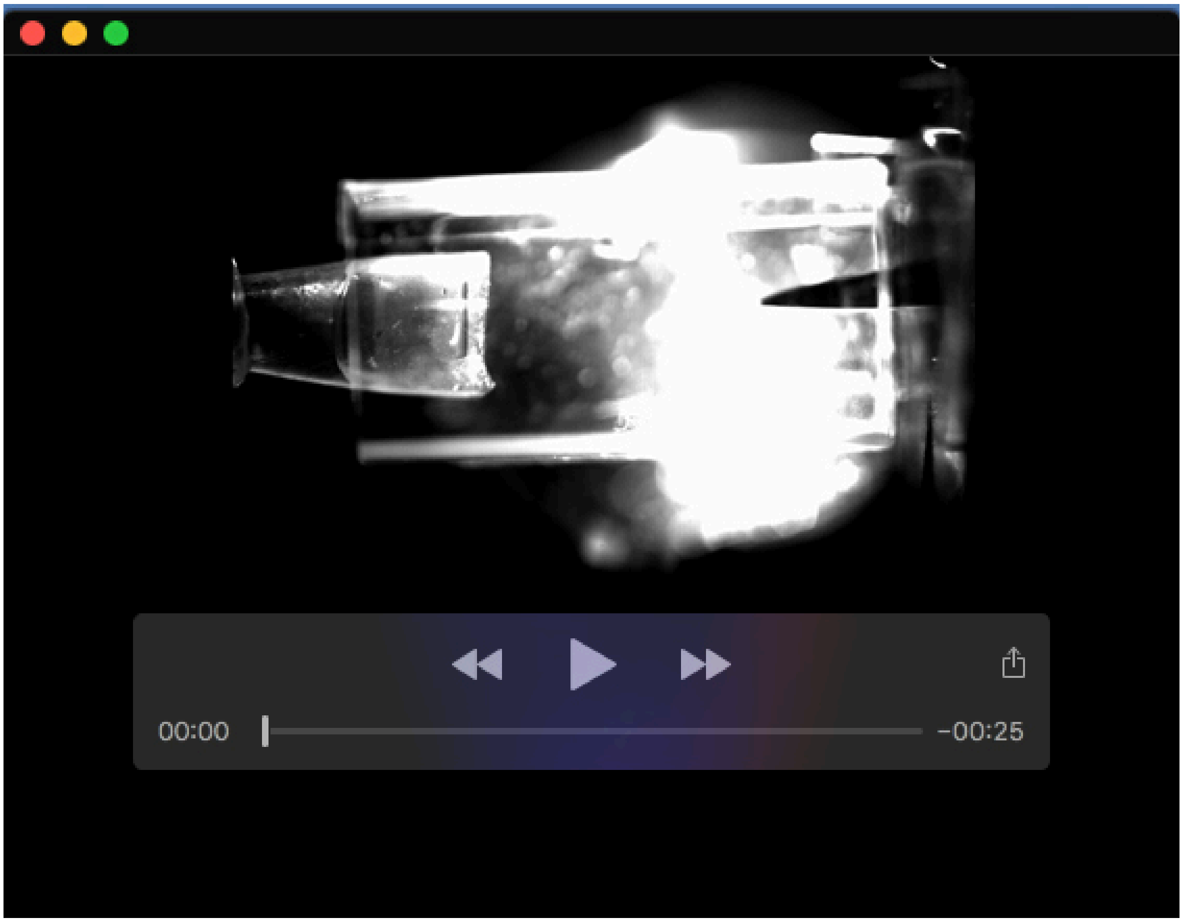

Movie 2. Dorsal view of a hummingbird drinking nectar. High-speed video (500 fps) of a Rufous-tailed hummingbird (*Amazilia tzacatl*), the nectar reservoir is located on the left. Note how the tongue tip splits inside the nectar, bending downwards and sideways; this is one of the reasons for using orthogonal angles in order to accurately track the displacement of the tongue. Achieving this backlit visualization through the keratin allowed us to track the nectar menisci inside the bill.

Journal of Experimental Biology • Supplementary information

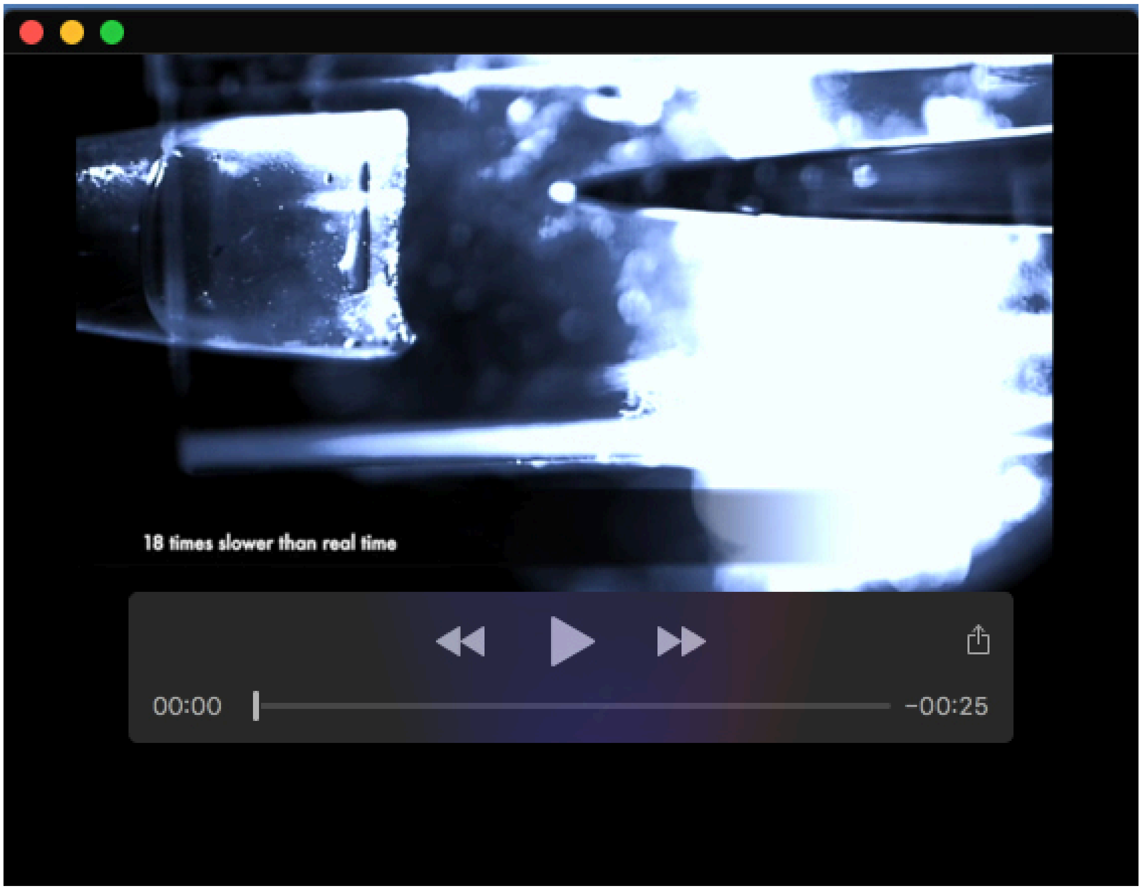

Movie 3. Edited footage of a hummingbird drinking nectar in dorsal view. High-speed video (500 fps) of a Rufous-tailed hummingbird (*Amazilia tzacatl*), the nectar reservoir is located on the left. First, the video was subject to monotonic enhancements in brilliance, contrast, and chroma, seeking to improve visualization of the tongue base. Second, the video is played back at different speeds in order to facilitate visual tracking of the tongue base and its interaction with the fluid inside the oral cavity. Third, we present an animated superimposition of mechanics data including shapes and shades obtained from the bill and tongue motion analyses (Fig. S2). Red line in the middle of the bill represents the tongue, and the V shape crossing the red line represents the tongue wings at its base. Blue shadow inside the bill depicts the nectar flowing intraorally. Green double-headed arrow on the left symbolizes the dorso-ventral separation of the bill tips, and yellow double-headed arrow on the right denotes the dorso-ventral separation of between maxilla and mandible at the bill base. Note that two licks are required to move a single aliquot of nectar from the nectar chamber to the throat.

Journal of Experimental Biology • Supplementary information
